# Jejunal Choristoma: A Very Rare Cause of Abdominal Pain in Children

**DOI:** 10.1155/2014/863647

**Published:** 2014-01-08

**Authors:** T. A. Olajide, S. O. Agodirin, R. W. Ojewola, O. O. Akanbi, T. O. Solaja, Johnson Oluremi Odesanya, O. O. Ariyibi

**Affiliations:** ^1^Paediatric Surgical Unit, Department of Surgery, Federal Medical Centre, PMB 201, Ido-Ekiti, Ekiti State, Nigeria; ^2^Department of Surgery, Ladoke Akintola University Teaching Hospital, Osogbo, Osun State, Nigeria; ^3^Department of Morbid Anatomy, Ladoke Akintola University Teaching Hospital, Osogbo, Osun State, Nigeria; ^4^Department of Anaesthesia, Federal Medical Centre, Ido-Ekiti, Ekiti State, Nigeria; ^5^Department of Morbid Anatomy, Federal Medical Centre, Owo, Ondo State, Nigeria

## Abstract

Choristoma is development of a normal tissue in an aberrant location. This report describes jejunal salivary choristoma (JSC) causing recurring episodes of abdominal discomfort in a 5-year-old girl. Exploratory laporatomy revealed a pale yellow subserosal jejunal lesion. Wedge resection of the lesion and repair of the bowel were performed. The child did well postoperatively and has since that time been free of pain at follow-up. Histopathological examination of the resected lesion revealed salivary gland choriostoma. Literature review (PUBMED search engine) revealed no previous report of this rare clinicopathologic entity. We conclude that choriostoma should be considered a possible differential when evaluating abdominal complaint in children.

## 1. Introduction

Abdominal pain is a common problem in children [1]. The presentation can be acute, chronic or, recurrent. The causes are diverse and some of the commonly suspected and diagnosed causes in our clinical practice include acute appendicitis, enteritis, symptomatic inguinal or umbilical hernias, intussusception, and helminthiasis [2]. When an organic cause of recurring abdominal pain is not found or diagnosed, the child is often labelled as having nonspecific abdominal pain, a diagnosis of exclusion. Rarely a diagnosis such as choristoma is considered preoperatively a cause of the abdominal symptoms in children.

Choriostoma is a form of heterotopia in which there is development of a normal tissue in an aberrant location [3]. The tissue grows at the same rate as the normal tissue and causes symptoms due to their pressure effect, ulceration, or malignant transformation. Choristomas generally are rare but they have been reported in the placenta [3]. In the gastrointestinal tract, a common site for choriostoma is in Meckel's diverticulum which is in itself a developmental anomaly [3] and the most common ectopic tissue in the gastro-intestinal tract is pancreatic tissues occurring mostly in the stomach, duodenum and proximal jejunum [4].

Salivary choriostoma is defined as a tumor - like growth of otherwise normal salivary gland found in an abnormal location. We present the ordeals and the difficulties of management of a young Nigerian girl who had salivary gland choriostoma in the jejunum presenting as a diagnostic dilemma.

## 2. Case Report

A 5-year-old female Nigerian child who was referred to our surgical facility on account of recurring abdominal pain. The pain was localized to the central abdomen, being colicky, aggravated by feeding and relieved occasionally by taking antacids. The episodes were frequent but erratic with the colics occurring mostly within 15 to 30 minutes of feeding; it was usually severe enough to stop her activities during the episode of the pain but she was a normal cheerful child in between the colics. She had occasional postprandial vomiting which sometimes relieved the symptoms. The vomitus contained only recently ingested meal and it did not contain blood. There was neither abdominal distention nor constipation. No history of diarrhoea, passage of worms, or haematochezia was found. There were neither fever, weight loss, nor anorexia. There was no groin swelling but she had a small umbilical protrusion which had been present since birth and had not changed in size even during the episodes of abdominal pain.

The frequent episodes either kept her away from school totally or prevented her from participating in the school activities. Before the onset of the episodic abdominal pains, she had never been operated upon, she had not been admitted to the hospital, and she had not been diagnosed of any chronic illness. Her genotype is AA. Both parents were anxious and they had taken her for review by several physicians on separate occasions including general surgeons and a paediatric surgeon.

On examination by all the physicians she visited, the only consistent positive abdominal finding was umbilical facial defect measuring 0.5 × 0.5 cm which was considered nonsignificant; otherwise she was a normal, well-nourished child. She was diagnosed as having mesenteric adenitis, gastroenteritis, helminthiasis, and peptic ulcer diseases at one time or another and had treatment for these varying diagnoses to no avail.

After several reviews and treatment with no finding of any organic disease, the assessment became non-specific abdominal pain with possibility of a child who is seeking attention. Psychotherapy was employed with no improvement in the clinical condition. Because of persistence of the symptoms, she had repair of the umbilical facial defect without abdominal exploration because this was the only possible diagnosis that had not been treated and could not be treated nonoperatively.

About two weeks after the repair of the umbilical defect, the symptoms resumed but now with the addition of hyperactive bowel sounds this led to a consideration of postoperative adhesions which further heightened the anxiety of the parents. Further evaluation with plain abdominal X-ray, abdominal ultrasound, gastrografin meal and follow-through showed no abnormalities. Upper gastrointestinal endoscopy and diagnostic laparoscopy were considered but were not done because there was no paediatric scope and there was no expertise for diagnostic laparoscopy at the time. Abdominal computer tomography was not done because the parents could not afford its cost. Eventually, a diagnostic exploratory laparotomy was done because of the continuing psychosocial and physical trauma suffered by the parents and the child.

At surgery, the bowel was examined from the duodeno jejunal junction to ileocecal junction. A pale yellow subserosal lesion was seen in the antimesenteric border of the jejunum about 45 cm from the duodeno-jejunal junction. There were enlarged mesenteric lymph nodes but there were neither adhesion nor other abnormalities. A wedge resection of the lesion was carried out and the bowel was repaired in a single layer using long-term absorbable sutures. The enlarged mesenteric nodes were also biopsied. She did well and was discharged home on the 6th post-operative day. The patient has been followed up serially in the clinic at one month, three months, six months, and a year after the procedure. She has been free of symptoms and has since that time been regular and participating fully in school activities.

## 3. Pathological Findings

**Figure 1:**
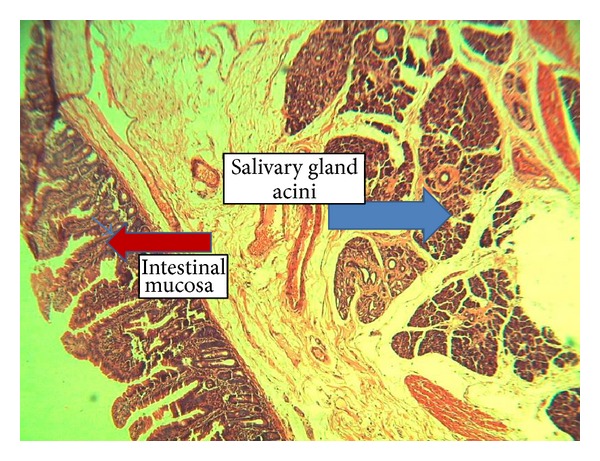
Salivary gland tissue in a background of intestinal tissue.

The specimen was a jejunal specimen measuring 1.9 × 1.2 cm with a grayish white mass measuring 0.9 × 0.6 cm and cut surface shows homogeneous tissue. Also a lymph node is included measuring 1.1 × 0.5 cm with a grayish white cut surface. Microscopic appearance shows a sub-mucosal tissue with lobules of serous glands with central lumen, reminiscent of salivary glands (Figure 1). The glands are composed of benign epithelial cells with regular nuclei and ample eosinophilic cytoplasm. The lymph node shows reactive follicular hyperplasia.

## 4. Discussion

A choriostoma is a rare congenital tumor-like lesion reported in human beings and other animal species consisting of histologically normal mature tissue located in an anatomic position different from its proper site [5]. Ectopic salivary gland has been reported in the head and neck regions but there is paucity of record of its presence in the gastrointestinal tract [6, 7]. Even though it was reported in the large intestine recently [8], there is no record of its presence in the jejunum. The index case constitutes a unique clinicopathologic entity in which clinical examinations and investigations could never have given a clue to the diagnosis and as such it constitutes a diagnostic dilemma in which the diagnosis can be made only after exploration and histopathological analysis of specimen.

Diagnosis and treatment of childhood acute abdominal pain can be straight forward if it has an organic cause and there is obvious need to intervene surgically. However, the scenario is usually complex when one cannot convincingly diagnose an organic cause clinically and radiologically and when patients have subacute presentation in which case it is referred to as non-specific abdominal pain. If indeed the cause of the pain is organic, the consideration of non-specific pain delays further investigations to reach a definitive diagnosis and treatment. This was clearly demonstrated in the case presented in this report, having consulted many physicians including general surgeons and a paediatric surgeon for a period of one year before presentation in our facility. The postprandial abdominal pain and the hyperactive bowel sound could be due to secretion of saliva by the glands in response to meal ingestion. This may increase the size of the gland, thereby causing some degree of luminal obstruction.

Most conditions diagnosed as non-specific pain in children eventually resolve with nonoperative management; however, if the symptoms persist and the psychosocial burden culminates as demonstrated in this case, the diagnosis may need to be reconsidered early and more aggressive steps in investigations or interventions may be required. Diagnostic laparoscopy would have been helpful in the index case because it could have resolved the problems with lesser risk of later adhesions. Theoretically, the diagnosis of a choriostoma might be reached pre-operatively using ultrasonographic techniques, computed tomography, or magnetic resonance imaging, but preoperative diagnosis of choriostoma is rarely possible regardless of improvements in diagnostic tools and techniques [9]. In situations where JSC is found at endoscopy, it should be resected in anticipation of its potential complications.

Another important lesson learnt from this presentation is that if the diagnosis is unclear and the decision is taken to operate on the child, then a full and thorough exploration should be carried out. If this had been done on the occasion of repair of the umbilical hernia, the diagnosis would have been reached and appropriate treatment could have been offered with possible complete resolution of the symptoms without a need for a second surgical intervention.

A delay in diagnosis and intervention may lead to the progression from partial to complete intestinal obstruction, ulceration with bleeding, or even malignant transformation. The malignant potential of such an ectopic tissue in such an unusual location cannot be overemphasized.

Generally, the prognosis of choriostoma depends on the location of the choriostoma. It is favourable in the case of a subcutaneous location because it can easily be removed surgically. A more extensive surgery may be required for lesions in the viscera when it becomes symptomatic or complicated.

We do not think that in a child with abdominal symptoms choriostoma will be considered a clinical diagnosis in the early periods of evaluation, neither do we think it is necessary to advocate that it should be at the top or near the top of the differentials. However, clinicians should be aware that this entity exists and it is worthy of consideration in a child presenting with bizarre abdominal complaint especially when all that seems to be common causes of the symptom pattern have been considered and ruled out.
